# Case Investigations of Infectious Diseases Occurring in Workplaces, United States, 2006–2015

**DOI:** 10.3201/eid2503.180708

**Published:** 2019-03

**Authors:** Chia-ping Su, Marie A. de Perio, Kristin J. Cummings, Anna-Binney McCague, Sara E. Luckhaupt, Marie Haring Sweeney

**Affiliations:** National Institute for Occupational Safety and Health, Cincinnati, Ohio, USA (C.-P. Su, M.A. de Perio, S.E. Luckhaupt, M.H. Sweeney);; Centers for Disease Control and Prevention, Atlanta, Georgia, USA (C.-P. Su, A.-B. McCague);; National Institute for Occupational Safety and Health, Morgantown, West Virginia, USA (K.J. Cummings, A.-B. McCague)

**Keywords:** communicable diseases, occupational illnesses, occupational exposure, epidemiologic studies, disease outbreaks, bacteria, viruses, fungi, parasites, National Institute for Occupational Safety and Health, United States, workplaces

## Abstract

Workers in specific settings and activities are at increased risk for certain infectious diseases. When an infectious disease case occurs in a worker, investigators need to understand the mechanisms of disease propagation in the workplace. Few publications have explored these factors in the United States; a literature search yielded 66 investigations of infectious disease occurring in US workplaces during 2006–2015. Reported cases appear to be concentrated in specific industries and occupations, especially the healthcare industry, laboratory workers, animal workers, and public service workers. A hierarchy-of-controls approach can help determine how to implement effective preventive measures in workplaces. Consideration of occupational risk factors and control of occupational exposures will help prevent disease transmission in the workplace and protect workers’ health.

Despite ongoing efforts to reduce, eliminate, and eradicate infectious diseases, infections continue to pose a global threat to human health. More than 2,000 years ago, Hippocrates noted that “there are many handicrafts and arts which cause those who exercise them certain… plagues” (*1*). Recent experiences with anthrax, severe acute respiratory syndrome (SARS), influenza A(H1N1), and Ebola virus disease have highlighted the importance of focusing on workplaces not only to identify at-risk populations but also to understand mechanisms of disease propagation and to implement successful control and prevention measures (*2*–*5*).

In the United States, work-related infectious diseases are identified in multiple ways. Workers, employers, or workplace health and safety offices may note an unusual case or cluster of disease. Local and state public health departments receive case reports of infectious disease from clinicians and laboratories, and they may conduct investigations, sometimes with assistance from the Centers for Disease Control and Prevention (CDC). CDC’s National Institute for Occupational Safety and Health (NIOSH) is the federal public health agency responsible for conducting research and making recommendations to prevent occupational safety and health risks, including work-related infectious diseases. The NIOSH Health Hazard Evaluation (HHE) program responds to requests from workers, employers, and public health agencies and conducts investigations of hazards including infectious diseases that occur in workplaces. Nongovernmental researchers also carry out investigations.

Investigators of work-related infectious disease must consider multiple factors related to the disease, workplace, and workers. Few publications have explored these factors. To illustrate the range of work-related infectious diseases that have been identified in the United States during 2006–2015 and to benefit future investigations, we examined the peer-reviewed literature and HHE reports on infectious diseases occurring in US workplaces. We describe occupational factors to consider and a systematic approach to control and prevent infectious disease in the workplace. We also note ways that specialized resources may be useful during the course of an investigation.

## Literature Review

### Methods

As defined by the World Health Organization, work-related diseases may have multiple causes, in which the work environment and other risk factors can play a role (https://www.who.int/occupational_health/activities/occupational_work_diseases/en). Work-related infectious diseases can be defined as those primarily caused by occupational exposure to biologic agents. These biologic agents, such as bacteria, fungi, viruses, and parasites, can be transmitted from human to human, from animal to human, or through environmental contact (*6*). We focused on studies of infectious disease occurring among workers in specific occupational groups or workplaces.

For the literature review, we followed the methods of Haagsma et al. (*6*). In March 2016, we searched PubMed for articles published since 2006. Our search strategy combined 3 groups of relevant keywords, including case investigation, workers/workplace/occupational, and infectious diseases. We also searched the NIOSH HHE reports database (https://www2a.cdc.gov/hhe/search.asp), using a similar strategy. We classified infectious diseases occurring in workplaces by industry category (work setting), using 2-digit codes of the North American Industry Classification System (NAICS) (https://www.census.gov/eos/www/naics), and by occupation (type of job). We describe detailed methods of our search of the literature and HHE reports in the Appendix.

### Results

The literature search yielded 67 articles and 7 HHE reports from 66 investigations of infectious diseases occurring in US workplaces during 2006–2015 (Table 1; Appendix). Cases were concentrated in specific industries and occupations, especially healthcare and work involving contact with animals, reflecting the potential for disease transmission from patients and animals. Complementing previous findings from a systematic review by Haagsma et al. (*6*), our review of worksite case investigations found that several work-related infectious disease outbreaks or individual cases have been reported among laboratory and public service workers.

**Table 1:** Reported case investigations of infectious disease occurring in workplaces, by industry categories, occupations, and diseases, United States, 2006–2015*

Industry category (NAICS code)	Occupations	Infectious diseases	References†
Agriculture, forestry, fishing, and hunting (11)	Hunter	Brucellosis	(*61*)
	Farmer	Variant influenza A(H3N2); *Escherichia coli* infection	(*83*); (*71*)
	Rodent breeder	LCMV infection	(*82*)
Construction (23)	Laborer	Coccidioidomycosis	(*23**,**25*)
Manufacturing (31–33)	Drum maker	Anthrax	(*2*)
	Poultry vaccine production worker	Salmonellosis	(*29*)
	Poultry-processing worker	Campylobacteriosis	(*17*)
	Furniture company worker	Tuberculosis	(*54*)
	Slaughterhouse inspector	Q fever	(*65*)
	Automobile manufacturing worker	Legionnaires’ disease	(*81*)
Transportation (48)	Truck driver	*Streptococcus suis* infection; cryptosporidiosis	(*79*); (*88*)
	Pilot, flight attendant	Malaria	(*37*)
Professional, scientific, and technical services (54)	Laboratory worker	Vaccinia virus infection, HIV infection, plague, cowpox, meningococcal disease, brucellosis	(*13**,**30**–**35**,86*)
Administrative support and waste management and remediation services (56)	Landscaper	Tularemia	(*21*)
Education services (61)	School employee, teacher	Influenza	(*8*)
Healthcare and social assistance (62)	Healthcare worker (security guard, nurse, nursing aide, physician, volunteer, environmental services)	Mumps; MRSA skin infection; norovirus gastroenteritis; adenovirus 14 infection; RSV infection; *Trichophyton tonsurans* skin infection; meningococcal disease; influenza; salmonellosis; Ebola virus disease; measles; TB	(*51*); (*52*); (*56*); (*57*); (*62*); (*64*); (*66*); (*11**,68*); (*87*); (*14*); (*92*); (*12**,77*)
	Childcare worker	*E. coli* infection	(*72*)
Arts, entertainment, and recreation (71)	Wildlife biologist	Plague	(*59*)
	Animal caretaker	MRSA skin infection	(*63*)
	Adult film performer	HIV infection	(*36*)
	Spa maintenance worker	MAC infection	(*22*)
	Filmmaker	Coccidioidomycosis	(*24*)
	Day camp counselor	Histoplasmosis	(*26*)
Food services (72)	Cook, food server	Norovirus gastroenteritis; salmonellosis; *E. coli* infection	(*20*); (*19*); (*89*)
Other services except public administration (81)	Embalmer	TB	(*16*)
	Animal refugee worker	Tuberculosis; sealpox virus infection	(*28*); (*18*)
	Pet store worker	Salmonellosis	(*74*)
	Missionary worker	Melioidosis; dengue fever	(*75*); (*70*)
Public administration (92)	US Customs officer	Measles	(*9**,**10*)
	Police officer	Meningococcal disease	(*66*)
	Firefighter	Cryptosporidiosis	(*88*)
	Correctional officer	Cryptosporidiosis; Shiga toxin–producing *E. coli* infection; TB; coccidioidomycosis;	(*78*); (*71*); (*12*); (*90*)
	Military	Legionellosis; TB	(*73*); (*53*)

*An expanded version of this table showing complete details on all cases is available online (https://wwwnc.cdc.gov/EID/article/25/3/18-0708-T1.htm). HIV, human immunodeficiency virus; LCMV, lymphocytic choriomeningitis virus; MAC, *Mycobacterium avium* complex; MRSA, methicillin-resistant *Staphylococcus aureus*; RSV, respiratory syncytial virus; TB, tuberculosis. NAICS, 2012 North American Industry Classification System (https://www.census.gov/eos/www/naics/). †Reference numbers >50 and additional details on the literature search are available in the Appendix.

Work-related cases were associated with a variety of infectious pathogens. Bacteria were responsible for most reported cases, followed by viruses, fungi, and parasites or protozoa. As noted previously (*6*), respiratory viruses and zoonotic pathogens still threaten workers’ health, especially for healthcare personnel and animal-contact workers. However, we also found reports of some emerging or reemerging pathogens, such as Ebola virus, lymphocytic choriomeningitis virus, norovirus, *Bacillus anthracis*, and *Yersinia pestis*, that caused several workplace disease clusters.

## Specific Considerations 

Many factors may combine to increase the risk for infection among workers during pathogen transmission. Categories of risk factors for work-related infections include disease factors (such as transmission mode), workplace factors, and worker factors. Considering each of these categories during case investigations is useful in planning and implementing prevention and control strategies.

### Disease Factors

Infectious disease can be transmitted via direct contact (including percutaneous), droplet, airborne (aerosol), vehicles (such as food, water, and fomites), and vectors (Table 2). The studies included in our review suggest that occupations involving interaction with the general population, particularly ill persons, pose an increased risk for infection.

**Table 2:** Reported case investigations of infectious disease pathogens occurring in workplaces, by mode of transmission and source of disease, United States, 2006–2015*

Mode of transmission	Disease (reference no.)†		
	Human	Animal	Environment
Direct contact (including percutaneous)	MRSA (*52,63*)	MRSA (*63*)	
	*Trichophyton tonsurans* (*64*)	*Bacillus anthracis* (*2*)	
	Ebola virus (*14**,**15*)	*Brucella* spp. (*61*)	
	HIV (*13**,**36*)	*Coxiella burnetii* (*65*)	
		*Yersinia pestis* (*59*)	
		Sealpox virus (*18*)	
		*Streptococcus suis* (*79*)	
		LCMV (*82*)	
		*Burkholderia pseudomallei* (*75*)	
Droplet	Adenovirus 14 (*57*)	Influenza virus (*83*)	
	influenza virus (*7**,**8**,**11**,68*)		
	*Neisseria meningitides* (*66*)		
	RSV (*62*)		
	Mumps virus (*51*)		
Airborne	*Mycobacterium tuberculosis* (*12**,**40**,53,54,77,85*)	*Mycobacterium tuberculosis* (*28*)	*Coccidioides* spp. (*23**–**25**,90*)
	Measles virus (*9**,**10**,92*)		MAC (*22*)
			*Legionella pneumophila* (*73,81*)
			*Francisella tularensis* (*21*)
			*Histoplasma capsulatum* (*26*)
Vehicles (fecal–oral)	Norovirus (*20**,56*)	*Salmonella* spp. (*19**,74*)	
	*E. coli* (*72*)	*E. coli* (*71,89*)	
		*Cryptosporidium* (*78,88*)	
		*Campylobacter jejuni (* *17* *)*	
Vectors		*Plasmodium* spp. (*37*)	
		Dengue virus (*70*)	

*LCMV, lymphocytic choriomeningitis virus; MAC, *Mycobacterium avium* complex; MRSA, methicillin-resistant *Staphylococcus aureus*; RSV, respiratory syncytial virus.  †Reference numbers >50 and additional details on the literature search are available in the Appendix.

As one example, teachers and public service workers may acquire respiratory virus infections, including influenza and measles, because their work may bring them in contact with persons who are ill (*7**–**10*). Workers in the healthcare industry are also at risk for influenza as well as airborne (such as tuberculosis [TB]) and percutaneously transmitted (such as HIV) infection from patients (*11**–**13*). In 2014, work-related Ebola virus infections among healthcare personnel were a substantial component of the Ebola epidemic worldwide; 2 healthcare personnel acquired Ebola virus disease within the United States (*14**,**15*). Occupational contact with human corpses can also result in infectious disease. In 2007, an embalmer in New York, New York, USA, contracted *Mycobacterium tuberculosis* from a cadaver (*16*).

Disease transmission patterns are also relevant to those whose work brings them in contact with animals, putting them at risk for zoonotic infections. Occupational exposure to livestock and poultry contributed substantially to work-related infectious diseases. Twenty-nine cases of *Campylobacter* infection occurred over a period of several years among workers at a poultry processing plant (*17*), and sealpox virus infections were reported among animal rescue workers in a marine mammal rehabilitation center (*18*).

Transmission of pathogens in the workplace may occur in 2 directions: workers can acquire infections in the workplace and then also may serve as vectors that spread the disease to others, such as clients and co-workers. We found that workers in food preparation and serving-related occupations have been identified as sources of transmission in foodborne outbreaks. Two delicatessen workers infected with *Salmonella* from occupational contact with chicken became the source of disease transmission in a 2007 salmonellosis cluster in Minnesota (*19*). Transmission of norovirus gastroenteritis among workers and customers at a restaurant has also been reported (*20*). These examples show that preventing workers from acquiring infections in workplaces may also prevent disease transmission among the general public.

Pathogens in the environment can also serve as a source of worker infections through the respiratory route. A 2006 report described a 21-year-old healthy landscaper diagnosed with tularemia. Traditionally thought of as a zoonotic pathogen, *Francisella tularensis* can also be acquired via aerosolized bacteria during occupational activities such as lawn mowing and leaf blowing (*21*). Another report described 2 spa maintenance workers infected with *Mycobacterium avium* complex organisms, which live in water and are highly resistant to disinfectants, such as chlorine. Occupational exposure to aerosolized bacteria during routine cleaning and maintenance of spa filters and tubs was the likely cause of this outbreak (*22*).

### Workplace Factors

Regardless of transmission mode, workplace factors can contribute to the propagation of infection. It is crucial to identify aspects of the workplace that pose biologic hazards, because those aspects may be amenable to controls. Such factors can include workplace characteristics, work practices and processes, and engineering and administrative issues. For example, work-related fungal respiratory infection is a concern in some areas. Outbreaks of coccidioidomycosis have occurred among construction workers and outdoor film production workers in California (*23**,**24*); during 2011–2014, a total of 44 cases of coccidioidomycosis were identified among workers constructing solar power farms (*25*). Outdoor workplaces with hot, dry conditions in areas of endemicity pose a risk for coccidioidomycosis because soil-disruptive activities and high winds expose workers to dust harboring *Coccidioides* spores. Other factors may also contribute. In 2012, Nebraska officials reported an investigation of a cluster of histoplasmosis among 32 day camp counselors (*26*). The probable infection source was campsite contamination of soil and picnic tables by bat guano, which probably became aerosolized during camp activities or cleanup.

Engineering factors can also promote disease transmission in the workplace. Previous studies have shown that workplace environmental characteristics contributed to *M. tuberculosis* transmission among workers (*27*). During the outbreak investigation of TB among workers at an elephant refuge in 2009, investigators found that shared air between the administrative area and the barn, along with pressure-washing of the barn by workers, contributed to transmission (*28*). Laboratories are another example of unique workplaces where engineering controls (such as biosafety cabinets and local exhaust ventilation) are essential for reducing or eliminating potential biologic hazards to workers. Laboratory workers have acquired *Salmonella* infection, vaccinia virus infection, plague, cowpox, brucellosis, meningococcal disease, and HIV infection through accidental direct contact with pathogens in the workplace over the past decade (*13*,*29**–**35*).

Administrative issues, including workplace policies and practices, can also play an important role in disease transmission in the workplace. In 2014, the California Department of Public Health reported an occupational HIV outbreak among adult film performers (*36*). Some adult film production companies rely on HIV testing results as a control and require performers to engage in penetrative sex without a condom. This approach is problematic, because during acute infection, a performer can transmit the infection even when HIV test results are negative. This example highlights that the lack of a protective administrative policy in the workplace could lead to infectious disease transmission among workers.

For workers in high-risk environments, employers have a responsibility to provide adequate prophylaxis for prevention of infectious disease. However, sometimes employers or workers are unfamiliar with or unable to comply with the relevant recommendations. For example, CDC staff found 4 malaria cases among employees of a commercial airline who had all traveled to Ghana and stayed at the same hotel before disease onset. None had used antimalarial chemoprophylaxis provided by the company (*37*). It is unclear why they did not use the chemoprophylaxis, but this cluster underscores the importance of a comprehensive malaria prevention program that includes education and counseling.

### Worker Factors

Individual characteristics, such as impaired immunity, inadequate prophylaxis, and socioeconomic and language factors, may increase the risk for acquisition and transmission of infectious diseases. For example, a case of fatal laboratory-acquired infection caused by *Y. pestis* occurred in a laboratorian in 2009 (*32*). No additional cases or major deficiencies in engineering controls were identified in this laboratory. A postmortem examination revealed that the affected worker had hereditary hemochromatosis, a condition that increases susceptibility to infection with certain bacterial pathogens. In another situation, *Campylobacter* infection among poultry-processing workers was found to occur most frequently during the first weeks of work, after which the workers develop immunity that may be protective against future infection (*17*). Therefore, investigators should consider that individual host susceptibility to certain diseases may play a role in disease transmission in workplaces.

Documented nosocomial transmission puts healthcare personnel at substantial risk for acquiring or transmitting several vaccine-preventable diseases, including hepatitis B, influenza, measles, mumps, rubella, pertussis, and varicella. The Advisory Committee on Immunization Practices and the Hospital Infection Control Practices Advisory Committee recommend that healthcare personnel be vaccinated or have documentation of immunity for all of these diseases; employers must formulate a comprehensive vaccination policy for all healthcare personnel (*38**,**39*). Workers in other settings, such as public services, may also be at risk for exposure to vaccine-preventable diseases, such as measles. In 2007, an airport officer contracted measles from an international traveler and may have transmitted it to a second airport worker (*9*). In 201l, a US Customs and Border Protection officer contracted measles after processing an arriving refugee with measles (*10*). All of these situations underscore that immunizations should be kept current for workers whose jobs involve frequent contact with the public.

In addition to individual susceptibility and immunity, a worker’s socioeconomic status could contribute to risk. Texas officials reported a TB outbreak investigation among workers in a meatpacking plant in 2011. The index case was in a foreign-born patient who had cavitary TB with acid-fast–positive smears. Most of the patient’s work contacts were foreign-born. Investigators found that low economic status, limited access to healthcare, and communication and language barriers caused delays in diagnosis and played significant roles in TB transmission among immigrant workers (*40*).

## Approach to Controlling Exposure to Protect Workers

Investigators of an infectious disease outbreak that is potentially related to a workplace must consider the “who, what, where, when, and why” questions of field epidemiology in the context of a unique and sometimes contentious environment. Stakeholders, including workers, employers, labor unions, trade associations, regulatory agencies, and members of the public, may have distinct and, at times, competing priorities. Barriers to implementing solutions may include those common to all epidemics, such as expense and time constraints, and others more specific to the occupational setting, such as a poorly developed safety culture.

To reduce worker exposure to potential occupational hazards, including biologic agents, we need feasible, effective measures that can be implemented in the workplace. Occupational health and safety specialists have long used the hierarchy of controls (Figure) to eliminate or minimize exposure to any occupational hazard. The control methods at the top of the graphic are potentially more effective and protective than those at the bottom. Here are examples of control recommendations made for identified occupational hazards using the hierarchy.

**Figure:**
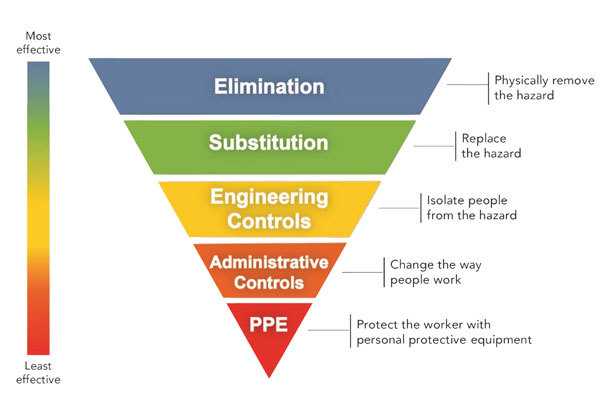
The hierarchy of controls for controlling exposures to occupational hazards. Source: NIOSH, https://www.cdc.gov/niosh/topics/hierarchy/default.html.

### Elimination and Substitution

Elimination and substitution are the most effective ways to reduce occupational hazards but sometimes can be difficult to implement in an existing work process. For infectious diseases, one should first assess the feasibility of not working in an area in which biohazards are present. If avoidance is not feasible, then decontaminating surfaces, items, and areas in a hazardous workplace can eliminate the possibility of transmission of infectious agents to workers. For environmentally associated laboratory infections, CDC has published guidance on methods for sterilization and disinfection in laboratories and on the levels of antimicrobial activity associated with liquid chemical germicides (*41*). However, employers and workers should also be aware of potential health risks when using such disinfectants (*42*).

Training on early recognition of the key signs and symptoms of common communicable diseases and the use of sick leave by workers may help to reduce disease transmission in the workplace. For example, CDC guidance for responding to influenza in schools during the 2009 influenza A(H1N1) pandemic included directing students and staff with influenza-like illness to stay home when ill (*43*). A recent study showed that offering paid sick leave to workers is likely to reduce the spread of disease in workplaces by increasing the rate at which sick workers stay home (*44*). However, the effectiveness of those measures still needs to be closely evaluated.

### Engineering Controls

Engineering controls in the field of occupational health and safety are physical changes to work processes or equipment to remove hazardous conditions or to place a barrier between workers and hazards. Engineering controls effectively protect workers without placing the primary responsibility of implementation on the worker. Ventilation is by far the most common engineering control, especially for airborne pathogens. Previous modeling data showed that the risk for TB infection in healthcare settings decreases exponentially as room ventilation rates increase (*45*). Thus, guidelines for preventing transmission of TB in healthcare settings recommend engineering controls such as local exhaust and general ventilation to prevent the spread and reduce the concentration of infectious droplet nuclei in the air. Supplementing ventilation systems with high-efficiency particulate air filtration, ultraviolet germicidal irradiation, or both can further enhance system performance and reduce the spread of airborne disease (*46*).

Nonventilation engineering controls also can reduce or eliminate pathogen exposure in the workplace. For example, dust suppression during construction, including continuous soil-wetting and proper covering, could decrease the risk for coccidioidomycosis among outdoor workers (*23*). In addition, evidence shows that engineering controls can reduce healthcare personnel’s exposure to bloodborne pathogens. Safety needle devices with built-in engineering controls reduce the risk for needlestick injury among healthcare personnel (*47*).

### Administrative Controls

Administrative controls are methods such as standard operating procedures that change the way work is performed. Their effectiveness depends on the availability of the control, employer commitment, and worker acceptance. Regular monitoring and reinforcement are necessary to ensure that workers follow policies and procedures consistently. The CDC publication Biosafety in Microbiological and Biomedical Laboratories addresses recommended practices for working safely from a biosafety perspective (*41*).

Developing and implementing a mandated workplace health regulation can be an effective administrative control. In December 1991, mandatory hepatitis B vaccination of all healthcare personnel (at employers’ expense) became a federal standard under the Occupational Safety and Health Act (*48*). The regulation accelerated the use of hepatitis B vaccine for healthcare personnel. As the result of routine vaccination and improved infection control precautions, the number of hepatitis B virus infections among healthcare personnel decreased from ≈10,000 in 1982 to ≈304 in 2004 (*39*). In addition, administrative measures of TB control, such as developing and implementing a written TB infection–control plan, prevent disease transmission in healthcare settings (*46*).

### Personal Protective Equipment

Various types of personal protective equipment (PPE) are available to minimize exposure to hazards in workplaces. PPE, such as respirators, gloves, goggles, and coveralls, provides a physical barrier between the worker and the infectious agent. Compared with other methods of controlling exposure, PPE is the least effective but probably best-known method used for infectious disease prevention. Proper use of PPE requires a comprehensive program and a high level of worker involvement and commitment. When engineering, work practice, and administrative controls are not feasible or do not provide sufficient protection, PPE may be the only reliable method of disease prevention for workers. Therefore, employers must provide their workers with PPE that is appropriate to the task and the correct size for the user, along with proper training on use and on donning and doffing methods.

In the early stages of epidemics of emerging infectious diseases, such as Ebola virus disease, lack or misuse of PPE can lead to infections in healthcare personnel. In 2014, the transmission of Ebola virus to 2 nurses who provided care to an Ebola-infected patient at a US hospital revealed the importance of directive PPE recommendations and standardized training efforts for healthcare personnel. Interventions included a system of trained observers supervising the donning and doffing of PPE (*5**,**49*). Even though the quality of evidence is low, the risk for contamination may be reduced by double-gloving, following directives for donning and doffing procedures, and instituting more active training (*50*).

## Additional Resources

The NIOSH Surveillance Program works with partners at CDC and the Council of State and Territorial Epidemiologists to promote inclusion of standard occupational information in the National Notifiable Disease Surveillance system (in which most conditions included are infectious) and in electronic medical records, to facilitate detection of possible disease transmission in workplaces (https://www.cdc.gov/niosh/topics/ehr/default.html). Once a disease cluster or outbreak is suspected, the NIOSH HHE program can be a resource for technical assistance and consultation during the case investigation in a workplace. Workers, employers, or public health professionals can request an evaluation of health hazards in the workplace (https://www.cdc.gov/niosh/hhe). NIOSH makes recommendations aimed at controlling the hazard; these are voluntary but can reduce risk and improve the health and safety of the workforce.

## Conclusions

We found that cases of work-related infectious diseases in the United States during 2006–2015 appeared to be concentrated in specific industries and occupations, especially in healthcare and among laboratory, animal, and public service workers. The biosafety programs in these industries could be strengthened. Case investigations of infectious disease occurring in a workplace can be challenging with regard to linking the symptoms of a disease to a specific pathogen or exposure source and identifying effective preventive strategies. A multidisciplinary approach that includes epidemiologists, physicians, industrial hygienists, and engineers may be beneficial.

Emerging and reemerging work-related infectious diseases will continue to threaten workers’ health. Previously published literature has identified several high-risk occupations, but other occupations may also be at risk. Indeed, we cannot know with certainty which industry or which workers will be at risk in the future. Considering occupational risk factors and controlling exposures among workers when investigating infectious disease may help prevent disease transmission in the workplace. In addition, because person-to-person transmission between workers and members of the public can propagate some disease outbreaks, assessment of worker infection may contribute to control of disease outbreaks in communities.

Appendix Additional information about infectious diseases in workplaces, United States, 2006–2015.
