# Supplementary material for: Case Investigations of Infectious Diseases Occurring in Workplaces, United States, 2006–2015
Source: Emerg Infect Dis. 2019 Mar;25(3):397–405. doi: 10.3201/eid2503.180708 (PMC6390751; doi:10.3201/eid2503.180708)
Supplement: Appendix — Additional information about infectious diseases in workplaces, United States, 2006–2015. [file 18-0708-Techapp-s1.pdf]

Article DOI: https://doi.org/10.3201/eid2503.180708

Case Investigations of Infectious Diseases Occurring in Workplaces, United States, 2006–2015

Appendix

Methods

We conducted a literature review by referring to the methods of Haagsma et al. (*6*).

In March 2016, with the assistance of a CDC librarian, we searched articles published since 2006 in the PubMed database (http://www.ncbi.nlm.nih.gov/PubMed). Our search strategy was to combine 3 groups of relevant keywords, including case investigation, workers/workplace, and infectious diseases. Below is the detailed search strategy:

(“outbreak” OR “cluster” OR “investigation” OR “epidemiology” OR “cases” OR “case report” OR “Notes”) AND (“Occupational diseases” OR “Environmental Exposure” OR “laborers” OR “occupational exposure” OR “workers” OR “employees” OR “workplace” OR “industry”) AND (“infectious diseases” OR “infection” OR “communicable disease” OR “Bacterial Infections” OR “viral infection” OR “pathogen” OR “zoonoses” OR “animals” OR Parasitic Diseases(Mesh)) AND eng(LA) AND United states(PL) NOT review(PT)

OR

(“occupational diseases”(mesh) and “infectious”(title) AND (“2006/01/01”(PDat): “2016/12/31”(PDat)) AND Humans(Mesh) AND English(lang))) OR (((((((“Occupational Exposure”(Mesh) AND Humans(Mesh) AND English(lang))) AND ((((((infectious or infection* or fungal or mycotic or mycoses or mycosis or viral or virus*)) OR ((((“Bacterial Infections and Mycoses”(Mesh)) OR “Viruses”(Mesh)) OR “Bacteria”(Mesh)) OR “Fungi”(Mesh) OR pathogen*))) AND (“occupational disease” OR “occupational diseases” OR laborer* OR “occupational exposure” OR “occupational exposures” OR worker* OR employee* OR workplace* OR industry OR industries) or (pathogen* and occupation*))) AND Humans(Mesh)

AND English(lang))) AND Humans(Mesh) AND English(lang))) AND (((((“Disease Outbreaks”(Mesh)) OR “Case Reports” (Publication Type)) OR (“Epidemiologic Studies”(Mesh) OR “Epidemiologic Study Characteristics as Topic”(Mesh))) OR (“Epidemiology”(Mesh) OR “epidemiology” (Subheading) OR “Molecular Epidemiology”(Mesh) OR “Bias (Epidemiology)”(Mesh) OR “Confounding Factors (Epidemiology)”(Mesh))) AND Humans(Mesh) AND English(lang))) AND (“2006/01/01”(PDat): “2016/12/31”(PDat)) AND Humans(Mesh) AND English(lang)) Filters: Publication date from 2006/01/01 to 2016/12/31; Humans; English

In addition, we searched NIOSH Health Hazard Evaluation (HHE) reports in the database (https://www2a.cdc.gov/hhe/search.asp) by using 2 filters:

1. Health effect: Viral, bacterial, parasitic, and fungal diseases.

2. Year published: 2006 to 2015.

We identified 2,402 articles that met the search criteria. After scanning the titles and/or abstracts, we excluded 2,045 for the following reasons:

1. Did not occur in the United States.

2. Not relevant to work-related infectious disease.

We also identified 40 HHE reports in the database and reviewed these along with the remaining articles. A total of 67 articles and 7 HHE reports from 66 infectious disease case investigations among workers were determined to be relevant to our topic, because each publication satisfied the following criteria:

1. It described a work-related infectious disease case investigation.

2. It mentioned one specific pathogen or disease.

3. It mentioned at least one specific occupational group or workplace.

4. It reported at least one symptomatic patient or seroconversion among workers.

5. The event occurred in the United States from 2006 to 2015.

The pathogens/diseases, industries (types of activity at the places of work), occupations (kinds of work), numbers of ill workers, transmission routes, and sources of exposure identified in this literature review are listed in Appendix Table 1.

Appendix Table 1. Data from reported case investigations of infectious diseases occurring in workplaces, United States, 2006–2015*

Year	State	Pathogen or disease	Industry (work setting)	Occupation	No. ill workers	Transmission mode†	Source of exposure†	References
2006	MI	Norovirus	Food services	Server, cook	15	Direct contact	Co-workers and/or patrons	(20)
2006	ME	Salmonellosis	Manufacturing (poultry vaccine production)	Unspecified employees in the same room	5 confirmed	Vehicles	Environment (lab)	(29)
2006	IL	Mumps	Healthcare (hospital)	Employees, not specified	7	Airborne	Patient	(51)
2006	RI	Methicillin-resistant Staphylococcus aureus (MRSA) soft-tissue infections	Healthcare (hospital)	Protective service (security guards)	5	Direct contact	Patient	(52)
2006	Unk	Tuberculosis	Public administration (Navy)	Sailors	139 latent TB	Airborne	Co-worker	(53)
2006	Mult	Tuberculosis (TB)	Manufacturing (furniture installation company)	Unspecified employees in the same building	42 latent TB	Airborne	Co-worker	(54)
2006	NM	Tularemia	Landscaping services	Landscaper	1	Aerosol or vectors	Animal (rabbits?) or environment	(21)
2007	NY	Tuberculosis	Funeral service	Embalmer	1	Airborne	Human corpse	(16)
2007	MN	Salmonellosis	Food services (grocery store delicatessen)	Food preparation	2	Vehicles	Animal (chicken)	(19)
2007	CA	Coccidioidomycosis	Construction (pipeline installation contractor)	Construction laborers	12	Airborne	Environment	(23, 55)
2007	MI	Measles	U.S. Customs	Federal airport officer	2	Airborne	International traveler	(9)
2007	OR	Norovirus	Healthcare (long-term residential treatment facility)	Healthcare (nurse, doctor, nursing aid, others)	242	Vehicles	Co-workers, patients, or visitors	(56)
2007	TX	Adenovirus 14	Healthcare (hospital)	Not specified healthcare workers	6	Droplet	Patient	(57)
2007	CT	Anthrax	Manufacturing	Drum maker	1	Direct contact	Animal (goat hide)	(2, 58)
2007	AZ	Plague	National Park Service	Wildlife biologist	1	Droplet	Animal (mountain lion carcass)	(59)
2005–2007	Mult	Vaccinia virus	Academic institution/ government facility	Laboratory workers	5	Direct contact	Environment (lab)	(30)
2008	MI	Norovirus	Food services	Dining service staff	3	Vehicles	Contaminated food?	(60)
2008	VA	Vaccinia virus	Academic institution	Laboratory worker	1	Direct contact	Environment (lab)	(31)
2008	Unk	Human immunodeficiency virus (HIV)	Laboratory	Technician	1	Needle puncture	Environment (lab)	(13)

Year	State	Pathogen or disease	Industry (work setting)	Occupation	No. ill workers	Transmission mode†	Source of exposure†	References
2007–2008	Mult	Brucellosis	Agriculture	Hunters	3	Direct contact	Animal (feral swine)	(61)
2007–2008	WA	Respiratory syncytial virus (RSV)	Healthcare (inpatient and outpatient cancer care)	Not specified healthcare workers	4	Droplet	Patients	(62)
2008	CA	MRSA skin infection	Zoo	Animal caretaker	20	Direct contact	Animal (elephant)	(63)
2009	IL	Plague	University	Laboratory worker	1	Direct contact	Environment (lab)	(32)
2009	NY	H1N1 influenza	High school	School employees	4	Droplet	Human	(7)
2009	MO	*Trichophyton tonsurans*	Healthcare	Staff, volunteer	21	Direct contact	Human	(64)
2009	Unk	Q fever	Manufacturing (slaughterhouse)	Inspector	1	Unknown	Animal (sheep and goat)	(65)
2009	CA	Meningococcal disease	Emergency response and healthcare	Police officer and respiratory therapist	2	Droplet	Patient	(66)
2009	HI	H1N1 influenza	School	Teacher	1	Droplet	Patient	(8)
2009	IL	H1N1 influenza	Hospital	Healthcare personnel (physician, nurse, other)	20	Droplet	Patient	(11)
2009	UT	H1N1 influenza	Hospital	Physicians	5	Droplet	Patient	(67, 68)
2009	TN	Tuberculosis	Elephant refuge	Employees (caregiver and administrative)	9 latent TB	Airborne	Animal (elephant)	(28, 69)
2009	NM	*Mycobacterium avium* complex	Spa	Maintenance worker	2	Airborne	Environment	(22)
2007–2009	Unk	Sealpox virus	Marine mammal rehabilitation facility	Marine mammal workers	2	Direct contact	Animal (seals or sea lions)	(18)
2010	Unk	Cowpox	Research lab	Lab worker	1	Direct contact	Environment (lab)	(33)
2010	FL, PA	Malaria	Commercial airline	Flight attendants and pilots	4	Vectors	?	(37)
2010	GA, NE	Dengue	Religious organizations	Missionary workers	7	Vectors	?	(70)
2010	CO	Shiga toxin–producing *E. coli*	Dairy farm	Employees at the dairy	10	Vehicles	Animal (cows)	(71)
2010	CO	Shiga toxin–producing *E. coli*	Child care center	Not specified staff	1 confirmed 11 suspect	Vehicles	Human	(72)
2010	Unk	Legionnaires' disease	Military base	Not specified base workers	7 confirmed 22 probable	Droplet	Environment	(73)
2010	Mult	Salmonellosis	Pet store	Employee	3	Vehicles	Animal (guinea pig)	(74)
2010	TX	Tuberculosis	Jail, hospital	Hospital and correctional staff	6 active TB	Airborne	Patient	(12)
2010	Unk	Melioidosis	Reptile importation and distribution center	Not specified	1	Direct contact	Animal (reptile)	(75)
2011	AZ	Tuberculosis	Hospital	Nursing assistant, nurse, medical support assistant, physician,	1 active TB 18 latent TB	Airborne	Patient	(76, 77)
2011	CA	Measles	U.S. Customs and Border Protection	Officer	1	Airborne	Patient (arriving refugee)	(10)
2011	IN, MI	Cryptosporidiosis	Fire department	Firefighter (response to a fire in a barn)	3	Vehicles	Animal (cattle)	(78)

Year	State	Pathogen or disease	Industry (work setting)	Occupation	No. ill workers	Transmission mode†	Source of exposure†	References
2011	MN	*Streptococcus* suis	Trucking company	Driver	1	Vehicles	Animal (swine)	(79)
2011	TX	Tuberculosis	Meat-packing plant	Not specified workers	20 latent TB	Airborne	Co-workers	(40)
2008–2011	VA	Campylobacterosis	Poultry-processing plant	Poultry workers	29	Vehicles	Animal (chicken)	(17, 80)
2008–2011	Mult	Brucellosis	University, public health lab	Laboratory workers	5	Direct contact	Environment (lab)	(34)
2009–2011	NY	Legionnaires' disease	Automobile and scrap metal shredding facility	Employees	4	Droplet	Environment	(81)
2012	CA	Coccidioidomycosis	Outdoor television-filming event	Cast or crew members	5 confirmed 5 probable	Airborne	Environment	(24)
2012	NE	Histoplasmosis	Day camp	Counselors	32	Airborne	Environment	(26)
2012	CA	Meningococcal disease	Research laboratory	Microbiologist	1	Direct contact	Environment (lab)	(35)
2012	IN, KY	Lymphocytic choriomeningitis virus (LCMV)	Rodent-breeding facilities	Employees	31	Direct contact	Animal	(82)
2012	OH	Variant influenza A(H3N2)	State fair	Swine exhibitor	20	Droplet	Animal (swine)	(83)
2012	AK	Tuberculosis	Long-term-care facility	Nursing assistant, environmental services	16 latent TB	Airborne	Resident/ patient	(84, 85)
2013	MA	Vaccinia virus	Research laboratory	Laboratory worker	1	Direct contact	Environment (lab)	(86)
2013	MN	Salmonellosis	Hospital	Phlebotomist	1	Vehicle	Patient	(87)
2013	KS	Cryptosporidiosis	Emergency responders	Law enforcement officers, tow truck employees	15	Vehicle	Animal (calves)	(88)
2013	PA	*E. coli* O157:H7	Restaurant	Food handlers: beef grinding	9	Vehicle	Animal (beef)	(89)
2009–2013	CA	Coccidioidomycosis	Prison	Employees	103	Airborne	Environment	(90, 91)
2014	TX	Ebola	Hospital	Nurses	2	Direct contact	Patient	(14)
2014	NY	Ebola	International medical humanitarian organization	Humanitarian aid worker	1	Direct contact	Patient	(15)
2014	CA	HIV	Adult-film production	Performers	2	Sexual contact	Co-worker	(36)
2011–2014	CA	Coccidioidomycosis	Construction (solar power farms)	Construction labors	44	Airborne	Environment	(25)
2015	AZ	Measles	Outpatient healthcare facility	Nurse	1	Airborne	Patient	(92)

*Unk, unknown; Mult, multiple.
†Suspected or confirmed.

Expanded Reference List

1. Price GM. Medicine and the industries. Bulletin of the American Academy of Medicine. 1917;18:134–9.

2. Centers for Disease Control and Prevention (CDC). Cutaneous anthrax associated with drum making using goat hides from West Africa—Connecticut, 2007. MMWR Morb Mortal Wkly Rep. 2008;57:628–31. PubMed

3. Fowler RA, Lapinsky SE, Hallett D, Detsky AS, Sibbald WJ, Slutsky AS, et al.; Toronto SARS Critical Care Group. Critically ill patients with severe acute respiratory syndrome. JAMA. 2003;290:367–73. PubMed http://dx.doi.org/10.1001/jama.290.3.367

4. Suarthana E, McFadden JD, Laney AS, Kreiss K, Anderson HA, Hunt DC, et al. Occupational distribution of persons with confirmed 2009 H1N1 influenza. J Occup Environ Med. 2010;52:1212–6. PubMed http://dx.doi.org/10.1097/JOM.0b013e3181fd32e4

5. Cummings KJ, Choi MJ, Esswein EJ, de Perio MA, Harney JM, Chung WM, et al. Addressing infection prevention and control in the first U.S. community hospital to care for patients with Ebola virus disease: context for national recommendations and future strategies. Ann Intern Med. 2016;165:41–9. PubMed http://dx.doi.org/10.7326/M15-2944

6. Haagsma JA, Tariq L, Heederik DJ, Havelaar AH. Infectious disease risks associated with occupational exposure: a systematic review of the literature. Occup Environ Med. 2012;69:140–6. PubMed http://dx.doi.org/10.1136/oemed-2011-100068

7. Lessler J, Reich NG, Cummings DA. New York City Department of Health and Mental Hygiene Swine Influenza Investigation Team. Outbreak of 2009 pandemic influenza A(H1N1) at a New York City school. N Engl J Med. 2009;361:2628–36.

8. Centers for Disease Control and Prevention (CDC). Outbreak of 2009 pandemic influenza A (H1N1) at a school—Hawaii, May 2009. MMWR Morb Mortal Wkly Rep. 2010;58:1440–4. PubMed

9. Chen TH, Kutty P, Lowe LE, Hunt EA, Blostein J, Espinoza R, et al. Measles outbreak associated with an international youth sporting event in the United States, 2007. Pediatr Infect Dis J. 2010;29:794–800. PubMed http://dx.doi.org/10.1097/INF.0b013e3181dbaacf

10. Centers for Disease Control and Prevention (CDC). Measles outbreak associated with an arriving refugee— Los Angeles County, California, August–September 2011. MMWR Morb Mortal Wkly Rep. 2012;61:385–9. PubMed

11. Magill SS, Black SR, Wise ME, Kallen AJ, Lee SJ, Gardner T, et al. Investigation of an outbreak of 2009 pandemic influenza A virus (H1N1) infections among healthcare personnel in a Chicago hospital. Infect Control Hosp Epidemiol. 2011;32:611–5. PubMed http://dx.doi.org/10.1086/660097

12. Medrano BA, Salinas G, Sanchez C, Miramontes R, Restrepo BI, Haddad MB, et al. A missed tuberculosis diagnosis resulting in hospital transmission. Infect Control Hosp Epidemiol. 2014;35:534–7. PubMed http://dx.doi.org/10.1086/675833

13. Joyce MP, Kuhar D, Brooks JT. Notes from the field: occupationally acquired HIV infection among health care workers—United States, 1985–2013. MMWR Morb Mortal Wkly Rep. 2015;63:1245–6. PubMed

14. Chevalier MS, Chung W, Smith J, Weil LM, Hughes SM, Joyner SN, et al.; Centers for Disease Control and Prevention (CDC). Ebola virus disease cluster in the United States—Dallas County, Texas, 2014. MMWR Morb Mortal Wkly Rep. 2014;63:1087–8. PubMed

15. Yacisin K, Balter S, Fine A, Weiss D, Ackelsberg J, Prezant D, et al.; Centers for Disease Control and Prevention (CDC). Ebola virus disease in a humanitarian aid worker—New York City, October 2014. MMWR Morb Mortal Wkly Rep. 2015;64:321–3. PubMed

16. Anderson JA, Meissner JS, Ahuja SD, Shashkina E, O'Flaherty T, Proops DC. Confirming *Mycobacterium tuberculosis* transmission from a cadaver to an embalmer using molecular epidemiology. Am J Infect Control. 2015;43:543–5. PubMed http://dx.doi.org/10.1016/j.ajic.2015.01.027

17. de Perio MA, Niemeier RT, Levine SJ, Gruszynski K, Gibbins JD. *Campylobacter* infection in poultry-processing workers, Virginia, USA, 2008–2011. Emerg Infect Dis. 2013;19:286–8. PubMed http://dx.doi.org/10.3201/eid1902.121147

18. Roess AA, Levine RS, Barth L, Monroe BP, Carroll DS, Damon IK, et al. Sealpox virus in marine mammal rehabilitation facilities, North America, 2007–2009. Emerg Infect Dis. 2011;17:2203–8. PubMed http://dx.doi.org/10.3201/eid1712.101945

19. Hedican E, Miller B, Ziemer B, LeMaster P, Jawahir S, Leano F, et al. Salmonellosis outbreak due to chicken contact leading to a foodborne outbreak associated with infected delicatessen workers. Foodborne Pathog Dis. 2010;7:995–7. PubMed http://dx.doi.org/10.1089/fpd.2009.0495

20. Centers for Disease Control and Prevention (CDC). Norovirus outbreak associated with ill food-service workers—Michigan, January–February 2006. MMWR Morb Mortal Wkly Rep. 2007;56:1212–6. PubMed

21. Hofinger DM, Cardona L, Mertz GJ, Davis LE. Tularemic meningitis in the United States. Arch Neurol. 2009;66:523–7. PubMed http://dx.doi.org/10.1001/archneurol.2009.14

22. Moraga-McHaley SA, Landen M, Krapfl H, Sewell CM. Hypersensitivity pneumonitis with *Mycobacterium avium* complex among spa workers. Int J Occup Environ Health. 2013;19:55–61. PubMed http://dx.doi.org/10.1179/2049396712Y.0000000015

23. Cummings KC, McDowell A, Wheeler C, McNary J, Das R, Vugia DJ, et al. Point-source outbreak of coccidioidomycosis in construction workers. Epidemiol Infect. 2010;138:507–11. PubMed http://dx.doi.org/10.1017/S0950268809990999

24. Wilken JA, Marquez P, Terashita D, McNary J, Windham G, Materna B; Centers for Disease Control and Prevention. Coccidioidomycosis among cast and crew members at an outdoor television filming event—California, 2012. MMWR Morb Mortal Wkly Rep. 2014;63:321–4. PubMed

25. Wilken JA, Sondermeyer G, Shusterman D, McNary J, Vugia DJ, McDowell A, et al. Coccidioidomycosis among workers constructing solar power farms, California, USA, 2011–2014. Emerg Infect Dis. 2015;21:1997–2005. PubMed http://dx.doi.org/10.3201/eid2111.150129

26. Centers for Disease Control and Prevention. Notes from the field: histoplasmosis outbreak among day camp attendees—Nebraska, June 2012. MMWR Morb Mortal Wkly Rep. 2012;21:747–8.

27. Davidow AL, Mangura BT, Wolman MS, Bur S, Reves R, Thompson V, et al. Workplace contact investigations in the United States. Int J Tuberc Lung Dis. 2003;7(Suppl 3):S446–52. PubMed

28. Murphree R, Warkentin JV, Dunn JR, Schaffner W, Jones TF. Elephant-to-human transmission of tuberculosis, 2009. Emerg Infect Dis. 2011;17:366–71. PubMed http://dx.doi.org/10.3201/eid1703.101668

29. Centers for Disease Control and Prevention. *Salmonella* serotype enteritidis infections among workers producing poultry vaccine—Maine, November–December 2006. MMWR Morb Mortal Wkly Rep. 2007;56:877–9. PubMed

30. Centers for Disease Control and Prevention. Laboratory-acquired vaccinia exposures and infections—United States, 2005–2007. MMWR Morb Mortal Wkly Rep. 2008;57:401–4. PubMed

31. Centers for Disease Control and Prevention. Laboratory-acquired vaccinia virus infection—Virginia, 2008. MMWR Morb Mortal Wkly Rep. 2009;58:797–800. PubMed

32. Centers for Disease Control and Prevention. Fatal laboratory-acquired infection with an attenuated *Yersinia pestis* strain—Chicago, Illinois, 2009. MMWR Morb Mortal Wkly Rep. 2011;60:201–5. PubMed

33. McCollum AM, Austin C, Nawrocki J, Howland J, Pryde J, Vaid A, et al. Investigation of the first laboratory-acquired human cowpox virus infection in the United States. J Infect Dis. 2012;206:63–8. PubMed http://dx.doi.org/10.1093/infdis/jis302

34. Traxler RM, Guerra MA, Morrow MG, Haupt T, Morrison J, Saah JR, et al. Review of brucellosis cases from laboratory exposures in the United States in 2008 to 2011 and improved strategies for disease prevention. J Clin Microbiol. 2013;51:3132–6. PubMed http://dx.doi.org/10.1128/JCM.00813-13

35. Sheets CD, Harriman K, Zipprich J, Louie JK, Probert WS, Horowitz M, et al. Fatal meningococcal disease in a laboratory worker—California, 2012. MMWR Morb Mortal Wkly Rep. 2014;63:770–2. PubMed

36. Wilken JA, Ried C, Rickett P, Arno JN, Mendez Y, Harrison RJ, et al. Occupational HIV transmission among male adult film performers—multiple states, 2014. MMWR Morb Mortal Wkly Rep. 2016;65:110–4. PubMed http://dx.doi.org/10.15585/mmwr.mm6505a3

37. Centers for Disease Control and Prevention. Notes from the field: malaria imported from West Africa by flight crews—Florida and Pennsylvania, 2010. MMWR Morb Mortal Wkly Rep. 2010;59:1412. PubMed

38. Bolyard EA, Tablan OC, Williams WW, Pearson ML, Shapiro CN, Deitchman SD; Hospital Infection Control Practices Advisory Committee. Guideline for infection control in healthcare personnel, 1998. Infect Control Hosp Epidemiol. 1998;19:407–63. PubMed http://dx.doi.org/10.2307/30142429

39. Advisory Committee on Immunization Practices; Centers for Disease Control and Prevention. Immunization of health-care personnel: recommendations of the Advisory Committee on Immunization Practices (ACIP). MMWR Recomm Rep. 2011;60(RR-7):1–45. PubMed

40. Kambali S, Nantsupawat N, Lee M, Nugent K. A workplace tuberculosis case investigation in the presence of immigrant contacts from high prevalence countries. J Community Health. 2015;40:576–80. PubMed http://dx.doi.org/10.1007/s10900-014-9946-3

41. CDC. Biosafety in microbiological and biomedical laboratories (BMBL), 5th edition [cited 2017 May 4]. https://www.cdc.gov/biosafety/publications/bmbl5

42. Quinn MM, Henneberger PK, Braun B, Delclos GL, Fagan K, Huang V, et al.; National Institute for Occupational Safety and Health (NIOSH), National Occupational Research Agenda (NORA) Cleaning and Disinfecting in Healthcare Working Group. Cleaning and disinfecting environmental surfaces in health care: Toward an integrated framework for infection and occupational illness prevention. Am J Infect Control. 2015;43:424–34. PubMed http://dx.doi.org/10.1016/j.ajic.2015.01.029

43. Centers for Disease Control and Prevention. CDC guidance for state and local public health officials and school administrators for school (K–12) responses to influenza during the 2009–2010 school year [cited 2017 May 4]. https://www.cdc.gov/h1n1flu/schools/schoolguidance.htm

44. Piper K, Youk A, James AE III, Kumar S. Paid sick days and stay-at-home behavior for influenza. PLoS One. 2017;12:e0170698. PubMed http://dx.doi.org/10.1371/journal.pone.0170698

45. Fennelly KP, Nardell EA. The relative efficacy of respirators and room ventilation in preventing occupational tuberculosis. Infect Control Hosp Epidemiol. 1998;19:754–9. PubMed http://dx.doi.org/10.2307/30141420

46. Jensen PA, Lambert LA, Iademarco MF, Ridzon R; CDC. Guidelines for preventing the transmission of *Mycobacterium tuberculosis* in health-care settings, 2005. MMWR Recomm Rep. 2005;54(RR-17):1–141. PubMed

47. Ballout RA, Diab B, Harb AC, Tarabay R, Khamassi S, Akl EA. Use of safety-engineered devices by healthcare workers for intravenous and/or phlebotomy procedures in healthcare settings: a systematic review and meta-analysis. BMC Health Serv Res. 2016;16:458. PubMed http://dx.doi.org/10.1186/s12913-016-1705-y

48. Bloodborne pathogens: the standard, 29 C.F.R. Sect. 1910.1030 (1991).

49. Hageman JC, Hazim C, Wilson K, Malpiedi P, Gupta N, Bennett S, et al. Infection prevention and control for Ebola in health care settings—West Africa and United States. MMWR Suppl. 2016;65:50–6. PubMed http://dx.doi.org/10.15585/mmwr.su6503a8

50. Verbeek JH, Ijaz S, Mischke C, Ruotsalainen JH, Mäkelä E, Neuvonen K, et al. Personal protective equipment for preventing highly infectious diseases due to exposure to contaminated body fluids in healthcare staff. Cochrane Database Syst Rev. 2016;4:CD011621. PubMed

51. Bonebrake AL, Silkaitis C, Monga G, Galat A, Anderson J, Trad JT, et al. Effects of mumps outbreak in hospital, Chicago, Illinois, USA, 2006. Emerg Infect Dis. 2010;16:426–32. PubMed http://dx.doi.org/10.3201/eid1603.090198

52. Patrozou E, Reid K, Jefferson J, Mermel LA. A cluster of community-acquired methicillin-resistant *Staphylococcus aureus* infections in hospital security guards. Infect Control Hosp Epidemiol. 2009;30:386–8. PubMed http://dx.doi.org/10.1086/596611

53. Centers for Disease Control and Prevention (CDC). Latent tuberculosis infection among sailors and civilians aboard U.S.S. Ronald Reagan—United States, January–July 2006. MMWR Morb Mortal Wkly Rep. 2007;55:1381–2. PubMed

54. Centers for Disease Control and Prevention (CDC). Workplace-based investigation of contacts of a patient with highly infectious tuberculosis—Maryland, District of Columbia, and Virginia, 2006. MMWR Morb Mortal Wkly Rep. 2008;57:94–8. PubMed

55. Das R, McNary J, Fitzsimmons K, Dobraca D, Cummings K, Mohle-Boetani J, et al. Occupational coccidioidomycosis in California: outbreak investigation, respirator recommendations, and surveillance findings. J Occup Environ Med. 2012;54:564–71. PubMed http://dx.doi.org/10.1097/JOM.0b013e3182480556

56. Centers for Disease Control and Prevention (CDC). Recurring norovirus outbreaks in a long-term residential treatment facility—Oregon, 2007. MMWR Morb Mortal Wkly Rep. 2009;58:694–8. PubMed

57. Yun HC, Prakash V. Transmission of adenovirus serotype 14 in the health care setting. Clin Infect Dis. 2008;46:1935–6. PubMed http://dx.doi.org/10.1086/588558

58. Nguyen TQ, Clark N, The 2006 NYC Anthrax Working Group. Public health and environmental response to the first case of naturally acquired inhalational anthrax in the United States in 30 years: infection of a New York City resident who worked with dried animal hides. J Public Health Manag Pract. 2010;16:189–200. PubMed http://dx.doi.org/10.1097/PHH.0b013e3181ca64f2

59. Wong D, Wild MA, Walburger MA, Higgins CL, Callahan M, Czarnecki LA, et al. Primary pneumonic plague contracted from a mountain lion carcass. Clin Infect Dis. 2009;49:e33–8. PubMed http://dx.doi.org/10.1086/600818

60. Centers for Disease Control and Prevention (CDC). Norovirus outbreaks on three college campuses—California, Michigan, and Wisconsin, 2008. MMWR Morb Mortal Wkly Rep. 2009;58:1095–100. PubMed

61. Centers for Disease Control and Prevention (CDC). *Brucella suis* infection associated with feral swine hunting—three states, 2007–2008. MMWR Morb Mortal Wkly Rep. 2009;58:618–21. PubMed

62. Chu HY, Englund JA, Podczervinski S, Kuypers J, Campbell AP, Boeckh M, et al. Nosocomial transmission of respiratory syncytial virus in an outpatient cancer center. Biol Blood Marrow Transplant. 2014;20:844–51. PubMed http://dx.doi.org/10.1016/j.bbmt.2014.02.024

63. Centers for Disease Control and Prevention (CDC). Methicillin-resistant *Staphylococcus aureus* skin infections from an elephant calf—San Diego, California, 2008. MMWR Morb Mortal Wkly Rep. 2009;58:194–8. PubMed

64. Shroba J, Olson-Burgess C, Preuett B, Abdel-Rahman SM. A large outbreak of *Trichophyton tonsurans* among health care workers in a pediatric hospital. Am J Infect Control. 2009;37:43–8. PubMed http://dx.doi.org/10.1016/j.ajic.2007.11.008

65. Towey A, Petitti C. OSHA compliance issues: a case of Q fever. J Occup Environ Hyg. 2009;6:D63–5. PubMed http://dx.doi.org/10.1080/15459620903152956

66. Centers for Disease Control and Prevention (CDC). Occupational transmission of *Neisseria meningitidis*—California, 2009. MMWR Morb Mortal Wkly Rep. 2010;59:1480–3. PubMed

67. de Perio MA, Brueck SE, Mueller CA. Evaluation of 2009 pandemic influenza A(H1N1) virus exposure among internal medicine housestaff and fellows. Health Hazard Evaluation Report, HETA 2009–0206–3117. October 2010 [cited 2017 May 4], https://www.cdc.gov/niosh/hhe/reports/pdfs/2009-0206-3117.pdf.

68. de Perio MA, Brueck SE, Mueller CA, Milne CK, Rubin MA, Gundlapalli AV, et al. Evaluation of 2009 pandemic influenza A(H1N1) exposures and illness among physicians in training. Am J Infect Control. 2012;40:617–21. PubMed http://dx.doi.org/10.1016/j.ajic.2012.01.014

69. Niemeier RT, Mead K, de Perio MA, Martin SB Jr, Burr GA. Evaluation of potential employee exposures to *Mycobacterium tuberculosis* at an elephant refuge. HHE Report No. 2010–0080–

3235. April 2015 [cited 2017 May 4], https://www.cdc.gov/niosh/hhe/reports/pdfs/2010-0080-3235.pdf

70. Centers for Disease Control and Prevention (CDC). Dengue virus infections among travelers returning from Haiti—Georgia and Nebraska, October 2010. MMWR Morb Mortal Wkly Rep. 2011;60:914–7. PubMed

71. Centers for Disease Control and Prevention (CDC). Outbreak of Shiga toxin-producing *Escherichia coli* O111 infections associated with a correctional facility dairy—Colorado, 2010. MMWR Morb Mortal Wkly Rep. 2012;61:149–52. PubMed

72. Brown JA, Hite DS, Gillim-Ross LA, Maguire HF, Bennett JK, Patterson JJ, et al. Outbreak of Shiga toxin–producing *Escherichia coli* serotype O26: H11 infection at a child care center in Colorado. Pediatr Infect Dis J. 2012;31:379–83. PubMed http://dx.doi.org/10.1097/INF.0b013e3182457122

73. Ambrose J, Hampton LM, Fleming-Dutra KE, Marten C, McClusky C, Perry C, et al. Large outbreak of Legionnaires' disease and Pontiac fever at a military base. Epidemiol Infect. 2014;142:2336–46. PubMed http://dx.doi.org/10.1017/S0950268813003440

74. Bartholomew ML, Heffernan RT, Wright JG, Klos RF, Monson T, Khan S, et al. Multistate outbreak of *Salmonella enterica* serotype enteritidis infection associated with pet guinea pigs. Vector Borne Zoonotic Dis. 2014;14:414–21. PubMed http://dx.doi.org/10.1089/vbz.2013.1506

75. Benoit TJ, Blaney DD, Gee JE, Elrod MG, Hoffmaster AR, Doker TJ, et al.; Centers for Disease Control and Prevention (CDC). Melioidosis cases and selected reports of occupational exposures to *Burkholderia pseudomallei*—United States, 2008–2013. MMWR Surveill Summ. 2015;64:1–9. PubMed

76. de Perio MA, Niemeier RT. Evaluation of exposure to tuberculosis among employees at a medical center—Arizona. Health Hazard Evaluation Report, HETA 2011–0137–3159. April 2012 [cited 2017 May 4], https://www.cdc.gov/niosh/hhe/reports/pdfs/2011-0137-3159.pdf

77. de Perio MA, Niemeier RT. Evaluation of exposure to tuberculosis among employees at a medical center. J Occup Environ Hyg. 2014;11:D63–8. PubMed http://dx.doi.org/10.1080/15459624.2014.888075

78. Centers for Disease Control and Prevention (CDC). Outbreak of cryptosporidiosis associated with a firefighting response—Indiana and Michigan, June 2011. MMWR Morb Mortal Wkly Rep. 2012;61:153–6. PubMed

79. Fowler HN, Brown P, Rovira A, Shade B, Klammer K, Smith K, et al. *Streptococcus suis* meningitis in swine worker, Minnesota, USA. Emerg Infect Dis. 2013;19:330–1. PubMed http://dx.doi.org/10.3201/eid1902.120918

80. de Perio MA, Gibbins JD, Niemeier RT. *Campylobacter* infection and exposures among employees at a poultry processing plant—Virginia. Health Hazard Evaluation Report, HETA 2011–0058–3157. April 2012 [cited 2017 May 4], https://www.cdc.gov/niosh/hhe/reports/pdfs/2011-0058-3157.pdf

81. Boylstein R, Bailey R, Piacitelli C, Schuler C, Cox-Ganser J, Kreiss K. Legionnaires' disease at an automobile and scrap metal shredding facility, New York. Health Hazard Evaluation Report, HETA 2011–0109–3162. August 2012 [cited 2017 May 4], https://www.cdc.gov/niosh/hhe/reports/pdfs/2011-0109-3162.pdf

82. Knust B, Ströher U, Edison L, Albariño CG, Lovejoy J, Armeanu E, et al. Lymphocytic choriomeningitis virus in employees and mice at multipremises feeder-rodent operation, United States, 2012. Emerg Infect Dis. 2014;20:240–7. PubMed http://dx.doi.org/10.3201/eid2002.130860

83. Greenbaum A, Quinn C, Bailer J, Su S, Havers F, Durand LO, et al. Investigation of an outbreak of variant influenza A(H3N2) virus infection associated with an agricultural fair—Ohio, August 2012. J Infect Dis. 2015;212:1592–9. PubMed http://dx.doi.org/10.1093/infdis/jiv269

84. de Perio MA, Niemeier RT. Evaluation of exposure to tuberculosis among employees at a long-term care facility. Health Hazard Evaluation Report No. 2012–0137–3178 May 2013. Revised June 2013 [cited 2017 May 4], https://www.cdc.gov/niosh/hhe/reports/pdfs/2012-0137-3178.pdf.

85. Jackson DA, Mailer K, Porter KA, Niemeier RT, Fearey DA, Pope L, et al. Challenges in assessing transmission of *Mycobacterium tuberculosis* in long-term-care facilities. Am J Infect Control. 2015;43:992–6. PubMed http://dx.doi.org/10.1016/j.ajic.2015.03.035

86. Hsu CH, Farland J, Winters T, Gunn J, Caron D, Evans J, et al.; Centers for Disease Control and Prevention (CDC). Laboratory-acquired vaccinia virus infection in a recently immunized person—Massachusetts, 2013. MMWR Morb Mortal Wkly Rep. 2015;64:435–8. PubMed

87. Centers for Disease Control and Prevention (CDC). Occupationally acquired *Salmonella* I 4,12:i:1,2 infection in a phlebotomist—Minnesota, January 2013. MMWR Morb Mortal Wkly Rep. 2013;62:525. PubMed

88. Webb LM, Tubach SA, Hunt DC; Centers for Disease Control and Prevention (CDC). Outbreak of cryptosporidiosis among responders to a rollover of a truck carrying calves—Kansas, April 2013. MMWR Morb Mortal Wkly Rep. 2014;63:1185–8. PubMed

89. Torso LM, Voorhees RE, Forest SA, Gordon AZ, Silvestri SA, Kissler B, et al. *Escherichia coli* O157:H7 outbreak associated with restaurant beef grinding. J Food Prot. 2015;78:1272–9. PubMed http://dx.doi.org/10.4315/0362-028X.JFP-14-545

90. de Perio MA, Niemeier RT, Burr GA. *Coccidioides* exposure and coccidioidomycosis among prison employees, California, United States. Emerg Infect Dis. 2015;21:1031–3. PubMed http://dx.doi.org/10.3201/eid2106.141201

91. de Perio MA, Burr GA. Evaluation of coccidioides exposures and coccidioidomycosis infections among prison employees. Health Hazard Evaluation Report No. 2013–0113–3198. January 2014 [cited 2017 May 4], https://www.cdc.gov/niosh/hhe/reports/pdfs/2013-0113-3198.pdf.

92. Jones J, Klein R, Popescu S, Rose K, Kretschmer M, Carrigan A, et al. Lack of measles transmission to susceptible contacts from a health care worker with probable secondary vaccine failure—Maricopa County, Arizona, 2015. MMWR Morb Mortal Wkly Rep. 2015;64:832–3. PubMed http://dx.doi.org/10.15585/mmwr.mm6430a5
